# Insights into Thermal Transport through Molecular π-Stacking

**DOI:** 10.1021/jacs.3c07921

**Published:** 2023-09-27

**Authors:** Ryosuke Takehara, Natsuki Kubo, Meguya Ryu, Suguru Kitani, Shusaku Imajo, Yoshiaki Shoji, Hitoshi Kawaji, Junko Morikawa, Takanori Fukushima

**Affiliations:** † Laboratory for Chemistry and Life Science, Institute of Innovative Research, Tokyo Institute of Technology, 4259 Nagatsuta, Midori-ku, Yokohama 226-8503, Japan; ‡ Department of Chemical Science and Engineering, School of Materials and Chemical Technology, Tokyo Institute of Technology, 4259 Nagatsuta, Midori-ku, Yokohama 226-8503, Japan; § National Methodology Institute of Japan (NMIJ), Advanced Industrial Science and Technology (AIST), Tsukuba Central 3, 1-1-1 Umezono, Tsukuba 305-8563, Japan; ∥ Laboratory for Materials and Structures, Institute of Innovative Research, Tokyo Institute of Technology, 4259 Nagatsuta-cho, Midori-ku, Yokohama 226-8503, Japan; ⊥ Institute for Solid State Physics, The University of Tokyo, Kashiwa, Chiba 277-8581, Japan; # Department of Materials Science and Engineering, School of Materials and Chemical Technology, Tokyo Institute of Technology, 2-12-1 Ookayama, Meguro-ku, Tokyo 152-8550, Japan; 7 Living Systems Materialogy (LiSM) Research Group, International Research Frontiers Initiative (IRFI), Tokyo Institute of Technology, 4259 Nagatsuta, Midori-ku, Yokohama 226-8503, Japan

## Abstract

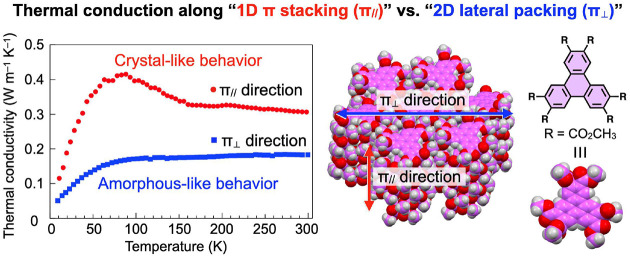

π-Stacking, which is a ubiquitous structural motif in assemblies of aromatic compounds, is well-known to provide a transport pathway for charge carriers and excitons, while its contribution to thermal transport is still unclear. Herein, based on detailed experimental observations of the thermal diffusivity, thermal conductivity, and specific heat of a single-crystalline triphenylene featuring a one-dimensionally π-stacked structure, we describe the nature of thermal transport through the π-stacked columns. We reveal that acoustic phonons are responsible for thermal transport through the π-stacked columns, which exhibit crystal-like behavior. Importantly, the thermal energy stored as intramolecular vibrations can also be transported by coupling to the acoustic phonons. In contrast, in the direction perpendicular to the π-stacked columns, an amorphous-like thermal transport behavior dominates. The present finding offers deep insight into nanoscale thermal transport in organic materials, where the constituent molecules exist as discrete entities linked together by weak intermolecular interactions.

## Introduction

An increased understanding of charge-carrier transport in organic materials has resulted in great advances in the molecular design of organic semiconductors, and organic electronics have been established,^1,2^ which inspire new concepts and uses for electronic devices developed conventionally using silicon technology.^3−6^ Following this excellent precedent, a deeper understanding of thermal transport through molecules in organic materials,^7,8^ which have classically been considered to be poor thermal conductors, could allow for the development of new thermal-management technologies.^9^ However, major difficulties still exist, since organic materials consist of molecules assembled together through weak intermolecular forces, and further, organic compounds are quite diverse, with unique structural anisotropies and properties. These are essential differences from metals and inorganic materials that feature continuous infinite structures of atoms linked together by strong chemical bonds. The science of thermal transport in organic materials has thus far been largely unexplored, and a first step toward a deeper understanding may be to focus on typical structural motifs and precisely evaluate the thermal conductivities present.

**Figure 1:**
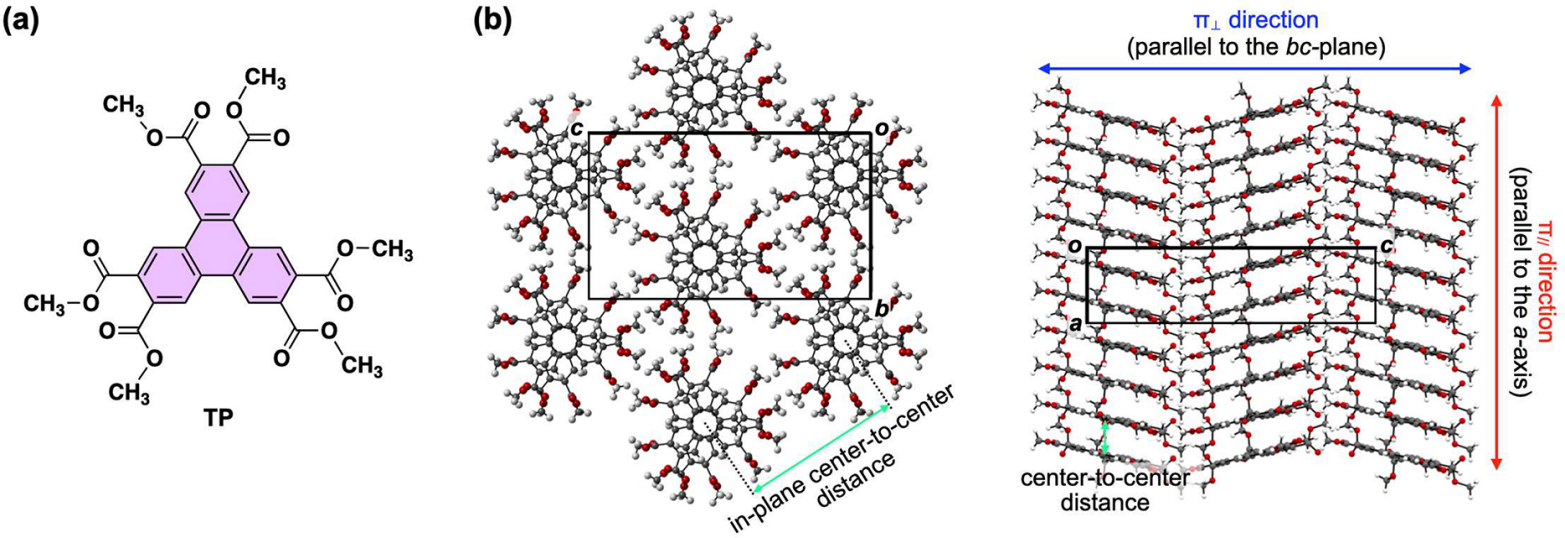
(a) Schematic structure of TP. (b) Single-crystal X-ray structure of TP,^11^ showing the intra- and intercolumnar center-to-center distances between π-stacked TP molecules. The red- (π_∥_) and blue-colored (π_⊥_) double-headed arrows represent the directions in which the thermal diffusivities were measured.

In this study, we addressed the issue of how π-stacking contributes to thermal transport. This packing motif, which is commonly seen in aromatic compounds,^10^ is well-known to serve as a transport pathway, typically for charge carriers and excitons, and serves as the origin of a variety of functions in π-conjugated molecular systems. However, it is still unclear whether π-stacked structures can offer an efficient thermal transport pathway. Herein, we focused on triphenylene-2,3,6,7,10,11-hexacarboxylic acid methyl ester (TP, Figure 1a),^11−14^ which we originally developed as a mesogenic motif for highly ordered discotic liquid crystals.^11^ This molecule forms a highly ordered, one-dimensional (1D) π-stacked columnar assembly with a plane-to-plane separation comparable to that of graphite (Figure 1b). Importantly, adjacent columns of TP in the crystal are segregated from each other by ester groups, which is favorable for the rigorous evaluation of 1D thermal transport in the π-stacking direction (Figure 1b). In addition, since planar π-conjugated molecules usually bear functional groups attached to their aromatic cores to improve solubility and processability, knowing the anisotropy of thermal transport through both π-stacking (intracolumnar) and functional groups (intercolumnar) could provide useful insights for the development of organic thermal-management materials.

**Figure 2:**
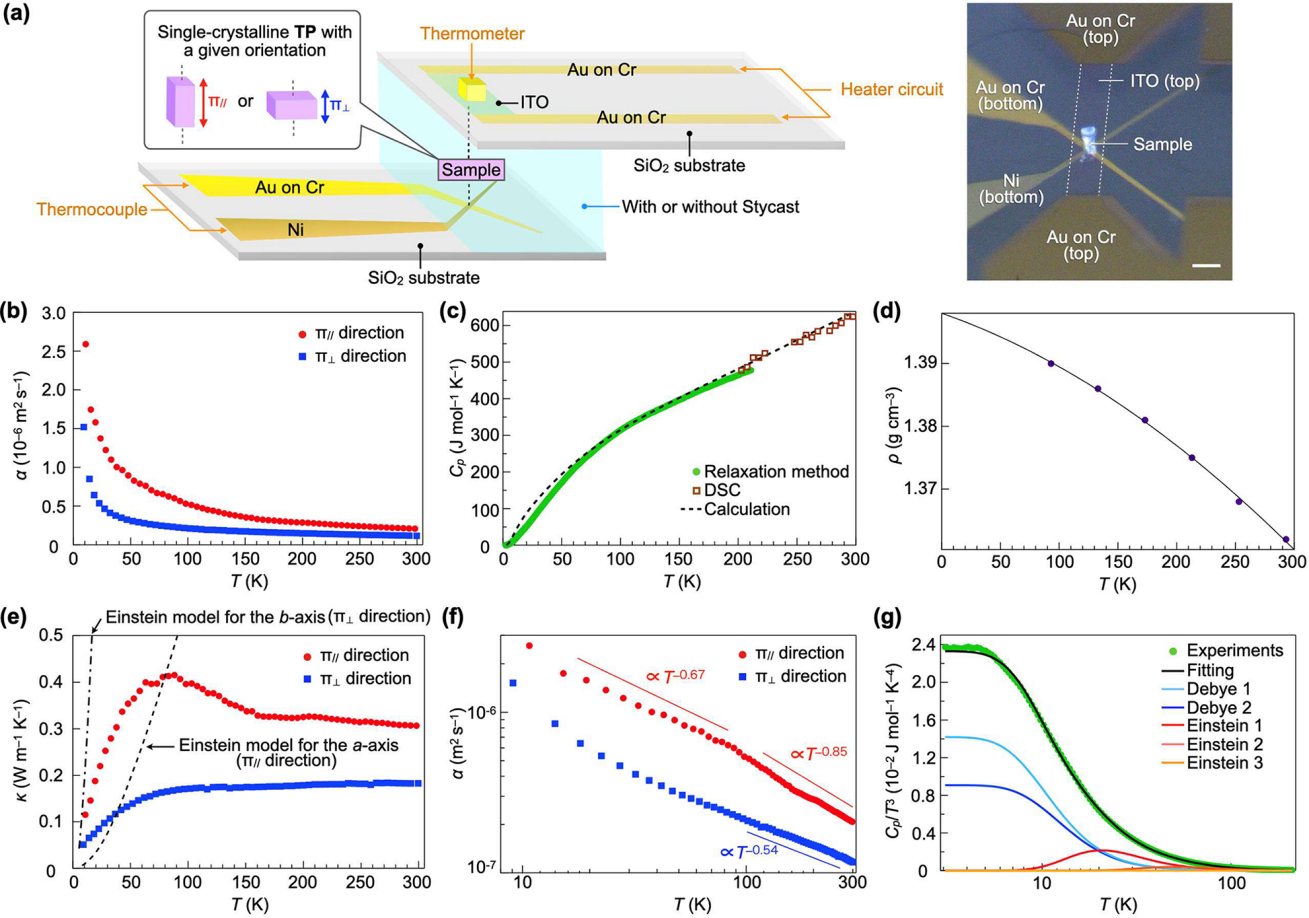
(a) Schematic illustration (left) and a photograph (right) of the experimental setup for μTWA measurements (for details, see the Supporting Information). At a given sample temperature (*T*), temperature waves are continuously input from a heater circuit to a sample, where the frequency (*f*) of temperature waves is chosen so that the number of temperature waves included within a sample range from 1 to 2, allowing for the accurate evaluation of thermal diffusivity at the sample temperature.^15−17^ A thermometer is attached to an ITO substrate with a heater circuit (Au) in such a way that it is positioned directly above the sample. When alternating current (AC) is applied to the heater circuit using a function generator while keeping the temperature rise of the thermometer below 1–2 K, the sample undergoes periodic Joule heating. The temperature change of the sample is detected as a change in voltage at the sensor. The phase delay (Δθ) between the input and the output detected by the sensor is plotted against *f*^1/2^. When a linear relationship between and *f*^1/2^ holds, e.g., Figure S1a, the slope gives the thermal diffusivity (α) of the sample. (b) Temperature dependence of the thermal diffusivity parallel (α_∥_) and perpendicular (α_⊥_) to the π-stacked columns of TP. (c) Temperature dependence of the specific heat (*C*_*p*_) of TP measured using the relaxation method (green filled circles) and DSC (brown open squares), along with those calculated using the Einstein and Debye specific heat models (black broken curve) (see also Figure S2a). (d) Temperature dependence of the density of TP obtained by single-crystal X-ray crystallography (see Table S2) and its extrapolation curve. (e) Temperature dependence of thermal conductivity in the directions parallel (κ_∥_) and perpendicular (κ_⊥_) to the π-stacked columns of TP, along with those calculated using the Einstein thermal conductivity model for *a*- and *b*-axes (see also Figure S2b). (f) Plots of ln α_∥_ and ln α_⊥_ against ln *T*. (g) Plots of experimentally obtained *C*_*p*_/*T*^3^ values and those calculated using the Einstein and Debye specific heat models, against *T*.

By means of a microtemperature wave analysis (μTWA) method,^15−17^ which enables the determination of thermal diffusivities in micrometer-sized materials, we investigated single-crystalline TP. In this method, by determining the crystallographic face in advance, the thermal diffusivities parallel (π_∥_) and perpendicular (π_⊥_) to the π-stacked columns of TP can be obtained independently (Figures 1b and 2a). The anisotropy of thermal conductivity (κ) in the corresponding directions was evaluated using the equation, κ = α*C*_*p*_ρ, where α, *C*_*p*_, and ρ are thermal diffusivity, specific heat at constant pressure, and density, respectively. Herein, based on an analysis of the temperature dependence of κ, α, and *C*_*p*_, we also discuss how thermal energy is transported parallel and perpendicular to the π-stacked columns.

## Results and Discussion

### Thermal Transport Properties of Single-Crystalline TP

As revealed by single-crystal X-ray analysis, the π-stacked columns of TP align in a direction parallel to the *a*-axis of the unit cell, i.e., perpendicular to the *bc*-plane (Figure 1b). μTWA measurements (Figure 2a) of single-crystalline TP at room temperature under an ambient atmosphere showed that the thermal diffusivity in the directions parallel (α_∥_) and perpendicular (α_⊥_) to the columns are 2.08 ± 0.23 × 10^–7^ and 1.23 ± 0.16 × 10^–7^ m^2^ s^–1^, respectively. Thus, the anisotropy (α_∥_/α_⊥_) is ca. 1.7. Figure 2b shows the temperature dependence of α_∥_ (red filled circles) and α_⊥_ (blue filled squares), measured under vacuum. Both α_∥_ and α_⊥_ increase monotonically with decreasing temperature, to give values of 2.59 ± 0.29 × 10^–6^ (at 11 K) and 1.52 ± 0.21 × 10^–6^ m^2^ s^–1^ (at 9 K) for α_∥_ and α_⊥_, respectively. The larger value of α_∥_ than α_⊥_ indicates that the intracolumnar mean free path (MFP) of thermal carriers is longer than the corresponding intercolumnar value. Although α_⊥_ abruptly increases below 30 K, overall, the rate of change is more pronounced for α_∥_ than for α_⊥_, suggesting that thermal carriers moving within the π-stacked columns are less likely to be scattered than those moving between them. To evaluate the thermal conductivity of single-crystalline TP, we measured the temperature dependence of its specific heat at constant pressure (*C*_*p*_) (Figure 2c) using the standard relaxation method and differential scanning calorimetry (DSC) in temperature ranges of 2–210 K (green filled circles) and 200–300 K (brown open squares), respectively,^18^ as well as its density (ρ) based on X-ray crystallography (Figure 2d). TP in the solid state shows no phase transitions in the temperature range measured. Upon cooling from 293 K, the density increased monotonically, and the value at 93 K was 2% higher than the initial value.

Figures 2e shows the temperature dependence of thermal conductivity for κ_∥_ (red filled circles) and κ_⊥_ (blue filled squares) obtained using the relationship κ = α*C*_*p*_ρ. At room temperature, κ_∥_ and κ_⊥_ are 0.31 ± 0.03 and 0.18 ± 0.03 W m^–1^ K^–1^, respectively. When the temperature is decreased, κ_∥_ increases, giving a maximum value at 80 K, and then decreases. This behavior is typical of systems in which acoustic phonons are responsible for carrying thermal energy, as is commonly observed for the thermal conductivity of electrically insulating crystalline materials.^19−25^ Such systems are known to undergo the Umklapp process, where collision and scattering between thermally excited phonons takes place to cause thermal resistance and shows an inverse relationship between thermal diffusivity and temperature.^26,27^ Indeed, in a temperature range of 300–70 K, α_∥_ is proportional to *T*^–0.85^–*T*^–0.99^, which is close to *T*^–1^ (Figure 2f; red filled circles, and Figure S3). A kink is seen around 70 K, and below that temperature, α_∥_ becomes proportional to *T*^–0.60^–*T*^–0.67^. Although the origin of this observation is unclear at the present, the phonon scattering process may be changed.

In contrast to the behavior of κ_∥_, κ_⊥_ monotonically decreases with decreasing temperature (Figure 2e). This trend is similar to the temperature dependence of thermal conductivity in crystalline polymers and amorphous materials.^28−33^ To be exact, amorphous materials exhibit temperature dependence with a concave portion in a certain temperature range due to Rayleigh scattering of phonons.^34−37^ Notably, α_⊥_ is proportional to *T*^–0.41^–*T*^–0.54^ even in the higher temperature range (Figure 2f; blue filled squares and Figure S3), in which α_∥_ is proportional to *T*^–0.85^–*T*^–0.99^. This behavior suggests that the dominant thermal carriers in the π_⊥_ direction could not be coherent phonons.

### Thermal Transport Properties of Single-Crystalline TP

According to the Dulong–Petit law (*C*/*n* = 3*R*, *R* = 8.314 J K^–1^ mol^–1^), the specific heat of crystalline materials is constant above their Debye temperatures (θ_D_). For inorganic materials consisting of atoms, *n* is defined as the number of moles of atoms, while for organic materials it can be represented as the number of moles of molecules. The behavior following the Dulong–Petit law can be understood by the Debye specific heat model per mol:
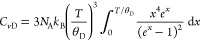
where *C*_*v*D_, *N*_A_, and *k*_B_ are the Debye specific heat at constant volume, the Avogadro constant, and the Boltzmann constant, respectively.^38^ According to this model, in the case of inorganic materials, thermal energy is stored as acoustic phonons originating from atomic motions with three translational degrees of freedom. In organic crystals, when a whole molecule is regarded as a single rigid-body, three translational (3*R*) and three rotational (3*R*) degrees of freedom need to be both considered. As a result, *C*_*v*_ can be represented by 6*R* above θ_D_.^39^ However, this alone does not explain the experimentally obtained *C*_*p*_ values of single-crystalline TP (Figure 2c; green filled circles and brown open squares). For example, *C*_*p*_ at 298 K was determined to be 624 J K^–1^ mol^–1^, which is nearly equal to 75*R*. Therefore, in addition to the six degrees of freedom assumed when the molecule is treated as a single rigid body, other degrees of freedom that arise from intramolecular vibrations of the total constituent atoms must be considered. In general, such localized vibrations can be interpreted by the Einstein specific heat model per degree of freedom,
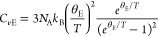
where *C*_*v*E_ and θ_E_ are Einstein specific heat at constant volume and Einstein temperatures.^40,41^

We found that the experimentally obtained *C*_*p*_ curve of single-crystalline TP can be reasonably explained by considering both Debye and Einstein specific heat models. As TP consists of 66 atoms, there are a total of 198 degrees of freedom. However, for the sake of simplicity, three translational and three rotational degrees of freedom were each treated with one Debye model equation (*C*_*v*Dtrans_ and *C*_*v*Drot_), while the remaining 192 degrees of freedom were treated with three Einstein specific heat models (*C*_*vEi*_, *C*_*vEj*_, and *C*_*vEk*_). Thus, the total specific heat (*C*_*v*_) of TP can be expressed as
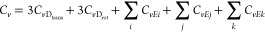
where *i* + *j* + *k* = 192. Provided that the difference between *C*_*p*_ and *C*_*v*_ is small,^42^ we can analyze the experimentally obtained *C*_*p*_ curve (Figure 2g; green filled circles) using this equation with θ_D_ and θ_E_ as parameters,^43^ and the best fit (Figure 2g; black curve) was obtained when the two Debye temperatures were 52 and 60 K and the three Einstein temperatures were 102, 247, and 1434 K.

Here, assuming that one longitudinal and two transverse acoustic phonon modes are all equivalent, phonon group velocity (*v*_ph_ave_) can be estimated using the equation
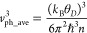
where ℏ and *n* are Dirac’s constant and the number density of molecules, respectively. When we used a crystallographically determined *n* value of, e.g., 1.45 × 10^27^ m^–3^ at 93 K, *v*_ph_ave_ values, as an average irrespective of the molecular alignment, are calculated to be 1528 and 1774 m s^–1^ from θ_D_ = 52 and 60 K, respectively. To verify the validity of these values, we carried out ultrasonic measurements (Figure S5), allowing us to evaluate longitudinal acoustic phonon group velocity (*v*_ph_π∥_) for a single-crystal sample of TP in the π_∥_ direction. The obtained *v*_ph_π∥_ (1900 ± 200 m s^–1^) roughly agreed with the values of *v*_ph_ave_. Since *v*_ph_π⊥_ could not be measured experimentally, the value (1527 m s^–1^) was determined so as to satisfy the relationship
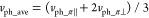
when the averaged values of 1528 and 1774 m s^–1^ were used as *v*_ph_ave_. This result demonstrates that in a low temperature range, the specific heat of organic crystals, in which discrete molecules weakly interact with each other by intermolecular forces to form a unit cell, can be interpreted by considering the Debye and Einstein specific heat models together, even if the huge numbers of degrees of freedom inherent to organic molecules are simplified in data treatment.

### Evaluation of Intramolecular Vibrations Based on the Einstein Thermal Conduction Model

In crystalline materials, except for metals, thermal energy stored as the Debye specific heat (6*R*) is carried by acoustic phonons. However, given that the *C*_*p*_ of single-crystalline TP corresponds to ∼75*R* at 298 K, most of it is interpreted in terms of Einstein specific heat. This means that thermal energy in the crystal is mainly stored as intramolecular vibrations. Since such localized vibrations rarely serve as thermal carriers, how thermal energy is transported in this crystal is an interesting question. The Einstein thermal conduction model that deals with thermal energy transport by the random-walk of localized vibrations may be useful to gain insight into this issue.^44^ Using this model, theoretical Einstein thermal conductivity (κ_*E*in_) of the present system can be obtained by the equation
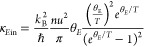
where *u* is the center-to-center intermolecular distance. Figure 2e (black broken curves) shows the Einstein thermal conductivity in the π_∥_ and π_⊥_ directions. However, the theoretical curves differ largely from the experimentally obtained curves, indicating that thermal energy does not propagate by localized intramolecular vibrations. This result agrees with the observation for an organic charge-transfer complex reported previously.^45^

**Figure 3:**
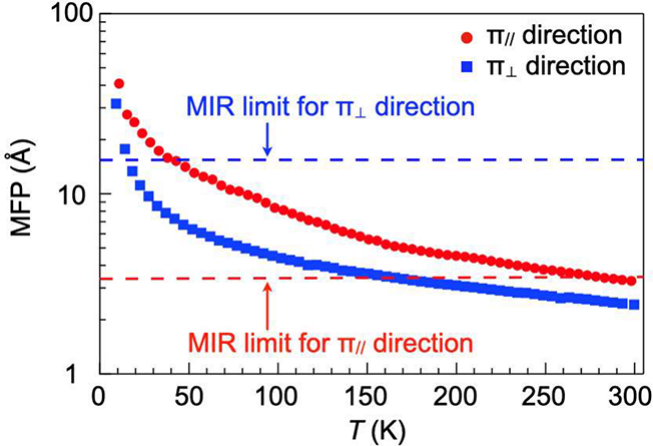
Temperature dependence of mean free paths and MIR limits in the π_∥_ and π_⊥_ directions.

To better understand the thermal transport properties, we determined the physical quantities of single-crystalline TP including the mean free path (MFP) of the thermal carriers. Based on the kinetic theory^46^ in which thermal carriers are regarded as gases, MFPs (*l*) are calculated by the equation
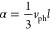
Figure 3 shows the temperature dependences of MFPs in the π_∥_ and π_⊥_ directions obtained using *v*_ph_π∥_ (1900 m s^–1^) and *v*_ph_π⊥_ (1527 m s^–1^), together with the Mott–Ioffe–Regel (MIR) limits (dashed lines). The MIR limit for crystalline inorganic materials is considered to approximately correspond to an interatomic distance.^47^ Here we define the MIR limits as the crystallographically determined center-to-center distances of TP molecules along the *a*- and *b*-axes in the π_∥_ and π_⊥_ directions, respectively (Figure 1b). If MFPs are higher than the MIR limits, then the scenario where acoustic phonons are responsible for thermal transport holds. Clearly, the MFP in the π_∥_ direction is larger than the MIR limit in almost all temperature ranges, indicating that acoustic phonons are responsible for thermal transport in the π_∥_ direction (Figure 3; red filled circles and red dashed line). For example, at 93 K, the MFP was calculated to be 8.92 ± 0.98 Å in the π_∥_ direction, which is higher than the MIR limit (3.40 Å). Even at 293 K, the MFP (3.19 ± 0.35 Å) is comparable to the MIR limit (3.46 Å). This is consistent with the experimentally observed temperature dependences of α_∥_ and κ_∥_ (Figure 2b,e), which are analogous to those of crystalline inorganic materials. On the other hand, the MFP in the π_⊥_ direction is much smaller than the MIR limit in almost all temperature ranges (Figure 3; blue filled squares and blue dashed line). Therefore, acoustic phonons rarely contribute to thermal transport in this direction, rationally explaining the amorphous-like behavior of the α_⊥_ and κ_⊥_ profiles.

With the above results in mind, we can discuss the thermal transport properties in the π_∥_ direction. Based on the degrees of freedom of TP, the specific heat originating from intramolecular vibrations plays a dominant role in the observed *C*_*p*_. Considering the relationship κ = α*C*_*p*_ρ, intramolecular vibrations should largely contribute to the thermal conduction in this system. However, attempts to understand the thermal transport properties of TP using the Einstein heat conduction model were unsuccessful, meaning that the localized intramolecular vibrations rarely carry thermal energy. Another important experimental finding is that the acoustic phonons are responsible for thermal transport in the π_∥_ direction. Thus, to address the question of how the thermal energy stored in intramolecular vibrations can be carried, we presume that the intramolecular vibrations could couple with the acoustic phonons, leading to thermal transfer in the crystal. Since the density of states (DOS) of acoustic phonons lies in the energy band below the Debye temperatures of the materials, the intramolecular vibrations with energies below the energy of the Debye temperatures of TP (52 and 60 K) are favorable for coupling with acoustic phonons. In the low temperature range, the energy of the thermally excited intramolecular vibrations is effectively transferred to the acoustic phonons, resulting in an increase in the DOS of the acoustic phonons and in turn the MFPs. This scenario does not hold true for the range above the Debye temperatures in which intramolecular vibrations with energies higher than the acoustic phonon energies dominate, and consequently, the MFPs decrease to the level of the MIR limit at around room temperature (Figure 3).

Meanwhile, we did not obtain any experimental evidence that supports the involvement of acoustic phonons in thermal transport in the π_⊥_ direction. The interactions that correlate molecules in this direction are only weak, nondirectional van der Waals forces between methyl ester side chains. In this situation, it is reasonable to assume that thermal conduction by acoustic phonons is unlikely,^48^ and therefore, the π_⊥_ direction does not provide an efficient pathway for transporting thermal energy stored in intramolecular vibrations.^49^ As a result, even though the entity is a single crystal, the π_⊥_ direction features an inherently amorphous nature in terms of thermal transport, which is manifested in its thermal conduction properties.

## Conclusions

Unlike inorganic crystals, in which atoms are linked together through strong covalent or ionic bonding to form infinite atomic chains, organic molecular assemblies are entities in which discrete molecules are linked together by weak intermolecular interactions. Herein, using single-crystalline TP featuring a highly ordered 1D π-stacked columnar assembly, we independently obtained the thermal conductivities parallel (κ_∥_) and perpendicular (κ_⊥_) to the π-stacked columns at room temperature as 0.31 ± 0.03 and 0.18 ± 0.03 W m^–1^ K^–1^, respectively. For comparison, graphite and WSe_2_, as representative materials having 2D atomic networks stacked into layered structures by van der Waals forces, show values 1 order of magnitude higher in the layer stacking direction (6.8 and 1.5 W m^–1^ K^–1^, respectively)^50,51^ at room temperature. This is most likely because the sizes of the 2D structural elements of these carbon/inorganic materials are considerably larger than that of TP.

To understand the thermal transport properties in organic materials, it is important to consider how the thermal energy stored in various vibrational modes within molecules is transported along with observing the contribution of acoustic phonons. As a general trend, organic materials with abundant intramolecular vibrations feature small MFPs, and their thermal diffusivities are on the order of 10^–7^ m^2^ s^–1^. Nonetheless, since few studies have hitherto been reported on thermal transport in organic crystals based on rigorous investigations into the anisotropy of molecular orientation and interaction modes, the contribution of acoustic phonons and intramolecular vibrations to the thermal transport properties remains unknown.

The present study focuses on π-stacking, which is a commonly observed motif in organic crystals, especially in systems that contain aromatic molecular units, and reveals the relationship between structural anisotropy and thermal transport properties. The experimental observations show that the acoustic phonons are responsible for the thermal conduction in the π-stacking direction, resulting in a temperature dependence characteristic of crystalline materials. On the other hand, in the direction perpendicular to the π-stacking, where no particular intermolecular interactions other than van der Waals forces operate, the thermal conduction behavior is amorphous. More specifically, the localized intramolecular vibrations hardly serve as a thermal carrier, but when coupled with acoustic phonons, they can be involved in thermal transport. At temperatures lower than the Debye temperature, this picture holds, while at higher temperatures, where the intramolecular vibrational energy is much larger than the Debye temperature, the coupling is suppressed, decreasing the mean free path. From these results, it is clear that efficient thermal transport in organic materials requires the connection of the constituent molecules by directional intermolecular interactions. This study provides deeper insight into the nature of thermal transport in organic materials and should encourage future efforts to rigorously investigate the relationship between other intermolecular interactions and thermal transport. The accumulation of these findings could lead to the development of unique thermal-management techniques based on organic materials.

## Supporting Information Available

Experimental details and methods including single-crystal X-ray analysis, specific heat measurements, thermal diffusivity measurements using a microtemperature wave analysis (μTWA), ultrasonic measurements, and density functional theory (DFT) calculations (PDF)

## References

[ref1] Brütting W. Physics of Organic Semiconductors; Wiley-VCH, 2005.

[ref2] Köhler A.; Bässler H. Electronic Processes in Organic Semiconductors: An Introduction; Wiley-VCH, 2015.

[ref3] Mitschke U.; Bäuerle P. The electroluminescence of organic materials. J. Mater. Chem. 2000, 10, 1471–1507. 10.1039/a908713c.

[ref4] Minemawari H.; Yamada T.; Matsui H.; Tsutsumi J.; Haas S.; Chiba R.; Kumai R.; Hasegawa T. Inkjet printing of single-crystal films. Nature 2011, 475, 364–367. 10.1038/nature10313. 21753752

[ref5] Chen L. X. Organic solar cells: recent progress and challenges. ACS Energy Lett. 2019, 4, 2537–2539. 10.1021/acsenergylett.9b02071.

[ref6] Matsuhisa N.; Chen X.; Bao Z.; Someya T. Materials and structural designs of stretchable conductors. Chem. Soc. Rev. 2019, 48, 2946–2966. 10.1039/C8CS00814K. 31073551

[ref7] Cui L.; Hur S.; Akbar Z. A.; Klöckner J. C.; Jeong W.; Pauly F.; Jang S. Y.; Reddy P.; Meyhofer E. Thermal conductance of single-molecule junctions. Nature 2019, 572, 628–633. 10.1038/s41586-019-1420-z. 31315129

[ref8] Fujii S.; Shoji Y.; Fukushima T.; Nishino T. Visualization of thermal transport properties of self-assembled monolayers on Au(111) by contact and noncontact scanning thermal microscopy. J. Am. Chem. Soc. 2021, 143, 18777–18783. 10.1021/jacs.1c09757. 34713695

[ref9] Moore A. L.; Shi L. Emerging challenges and materials for thermal management of electronics. Mater. Today 2014, 17, 163–174. 10.1016/j.mattod.2014.04.003.

[ref10] Hunter C. A.; Sanders J. K. M. The nature of π-π interactions. J. Am. Chem. Soc. 1990, 112, 5525–5534. 10.1021/ja00170a016.

[ref11] Osawa T.; Kajitani T.; Hashizume D.; Ohsumi H.; Sasaki S.; Takata M.; Koizumi Y.; Saeki A.; Seki S.; Fukushima T.; Aida T. Wide-range 2D lattice correlation unveiled for columnarly assembled triphenylene hexacarboxylic esters. Angew. Chem., Int. Ed. 2012, 51, 7990–7993. 10.1002/anie.201203077. 22753399

[ref12] Kajitani T.; Motokawa K.; Kosaka A.; Shoji Y.; Haruki R.; Hashizume D.; Hikima T.; Takata M.; Yazawa K.; Morishima K.; Shibayama M.; Fukushima T. Chiral crystal-like droplets displaying unidirectional rotational sliding. Nat. Mater. 2019, 18, 266–272. 10.1038/s41563-018-0270-7. 30664694

[ref13] Shoji Y.; Kobayashi M.; Kosaka A.; Haruki R.; Kumai R.; Adachi S.; Kajitani T.; Fukushima T. Design of discotic liquid crystal enabling complete switching along with memory of homeotropic and homogeneous alignment over a large area. Chem. Sci. 2022, 13, 9891–9901. 10.1039/D2SC03677K. 36128239 PMC9430577

[ref14] Shoji Y.; Komiyama R.; Kobayashi M.; Kosaka A.; Kajitani T.; Haruki R.; Kumai R.; Adachi S.; Tada T.; Karasawa N.; et al. Collective bending motion of a two-dimensionally correlated bowl-stacked columnar liquid crystalline assembly under a shear force. Sci. Adv. 2023, 9, eadg8202 10.1126/sciadv.adg8202. 37172082 PMC10181172

[ref15] Morikawa J.; Hashimoto T. Thermal diffusivity of aromatic polyimide thin films by temperature wave analysis. J. Appl. Phys. 2009, 105, 113506 10.1063/1.3116509.

[ref16] Ryu M.; Takamizawa S.; Morikawa J. Thermal diffusivity of organosuperelastic soft crystals during stress-induced phase transition. Appl. Phys. Lett. 2021, 119, 251902 10.1063/5.0055707.

[ref17] Morikawa J.; Orie A.; Hashimoto T.; Juodkazis S. Thermal and optical properties of the femtosecond-laser-structured and stress-induced birefringent regions in sapphire. Opt. Express 2010, 18, 8300 10.1364/OE.18.008300. 20588676

[ref18] The relaxation method is used to determine the specific heat from the relaxation time when heat accumulated in the sample escapes. In the relaxation method, a pellet sample prepared by compression molding of single crystals was used. Precisely, for organic materials featuring low thermal conductivity, the values obtained are likely dependent on the sample thickness, particularly for measurements at high temperatures. This is because the heat capacity is larger at high temperatures, and it takes a longer time for heat to escape from the sample if the sample is thick, resulting in a temperature distribution within the sample. Thus, we used the value obtained by DSC for *C*_*p*_ above 200 K.

[ref19] Ho C. Y.; Powell R. W.; Liley P. E. Thermal conductivity of the elements. J. Phys. Chem. Ref. Data 1972, 1, 279–421. 10.1063/1.3253100.

[ref20] Slack G. A. Nonmetallic crystals with high thermal conductivity. J. Phys. Chem. Solids 1973, 34, 321–335. 10.1016/0022-3697(73)90092-9.

[ref21] Slack G. A.; Tanzilli R. A.; Pohl R. O.; Vandersande J. W. The intrinsic thermal conductivity of AIN. J. Phys. Chem. Solids 1987, 48, 641–647. 10.1016/0022-3697(87)90153-3.

[ref22] Cahill D. G.; Pohl R. O. Lattice vibrations and heat transport in crystals and glasses. Annu. Rev. Phys. Chem. 1988, 39, 93–121. 10.1146/annurev.pc.39.100188.000521.

[ref23] Okada Y.; Uno M.; Nakazawa Y.; Sasai K.; Matsukawa K.; Yoshimura M.; Kitaoka Y.; Mori Y.; Takeya J. Low-temperature thermal conductivity of bulk and film-like rubrene single crystals. Phys. Rev. B 2011, 83, 113305 10.1103/PhysRevB.83.113305.

[ref24] Langenberg E.; Ferreiro-Vila E.; Leborán V.; Fumega A. O.; Pardo V.; Rivadulla F. Analysis of the temperature dependence of the thermal conductivity of insulating single crystal oxides. APL Mater. 2016, 4, 104815 10.1063/1.4966220.

[ref25] Inyushkin A. V.; Taldenkov A. N.; Ralchenko V. G.; Bolshakov A. P.; Koliadin A. V.; Katrusha A. N. Thermal conductivity of high purity synthetic single crystal diamonds. Phys. Rev. B 2018, 97, 144305 10.1103/PhysRevB.97.144305.

[ref26] Callaway J. Model for lattice thermal conductivity at low temperatures. Phys. Rev. 1959, 113, 1046–1051. 10.1103/PhysRev.113.1046.

[ref27] Holland M. G. Analysis of lattice thermal conductivity. Phys. Rev. 1963, 132, 2461–2471. 10.1103/PhysRev.132.2461.

[ref28] Cahill D. G.; Pohl R. O. Thermal conductivity of amorphous solids above the plateau. Phys. Rev. B 1987, 35, 4067–4073. 10.1103/PhysRevB.35.4067. 9941934

[ref29] Cahill D. G.; Pohl R. O. Heat flow and lattice vibrations in glasses. Solid State Commun. 1989, 70, 927–930. 10.1016/0038-1098(89)90630-3.

[ref30] Cahill D. G.; Watson S. K.; Pohl R. O. Lower limit to the thermal conductivity of disordered crystals. Phys. Rev. B 1992, 46, 6131–6140. 10.1103/PhysRevB.46.6131. 10002297

[ref31] Shrestha R.; Li P.; Chatterjee B.; Zheng T.; Wu X.; Liu Z.; Luo T.; Choi S.; Hippalgaonkar K.; de Boer M. P.; Shen S. Crystalline polymer nanofibers with ultra-high strength and thermal conductivity. Nat. Commun. 2018, 9, 1664 10.1038/s41467-018-03978-3. 29695754 PMC5916895

[ref32] Zhang Y.; Zhang X.; Yang L.; Zhang Q.; Fitzgerald M. L.; Ueda A.; Chen Y.; Mu R.; Li D.; Bellan L. M. Thermal transport in electrospun vinyl polymer nanofibers: effects of molecular weight and side groups. Soft Matter 2018, 14, 9534–9541. 10.1039/C8SM01696H. 30376032

[ref33] Kim T.; Drakopoulos S. X.; Ronca S.; Minnich A. J. Origin of high thermal conductivity in disentangled ultra-high molecular weight polyethylene films: ballistic phonons within enlarged crystals. Nat. Commun. 2022, 13, 2452 10.1038/s41467-022-29904-2. 35508468 PMC9068786

[ref34] Zeller R. C.; Pohl R. O. Thermal conductivity and specific heat of noncrystalline solids. Phys. Rev. B 1971, 4, 2029–2041. 10.1103/PhysRevB.4.2029.

[ref35] Raychaudhuri A. K. Origin of the plateau in the low-temperature thermal conductivity of silica. Phys. Rev. B 1989, 39, 1927–1931. 10.1103/PhysRevB.39.1927. 9948412

[ref36] Olson J. R.; Pohl R. O.; Vandersande J. W.; Zoltan A.; Anthony T. R.; Banholzer W. F. Thermal conductivity of diamond between 170 and 1200 K and the isotope effect. Phys. Rev. B 1993, 47, 14850–14856. 10.1103/PhysRevB.47.14850. 10005859

[ref37] Feldman J. L.; Kluge M. D.; Allen P. B.; Wooten F. Thermal conductivity and localization in glasses: Numerical study of a model of amorphous silicon. Phys. Rev. B 1993, 48, 12589–126002. 10.1103/PhysRevB.48.12589. 10007627

[ref38] Debye P. On the theory of specific heats. Ann. Phys. 1912, 344, 789–839. 10.1002/andp.19123441404.

[ref39] Venkataraman G.; Sahni V. C. External vibrations in complex crystals. Rev. Mod. Phys. 1970, 42, 409–470. 10.1103/RevModPhys.42.409.

[ref40] Einstein A. Planck’s theory of radiation and the theory of heat capacity of solids. Ann. Phys. 1907, 327, 180–190. 10.1002/andp.19063270110.

[ref41] Fulem M.; Laštovka V.; Straka M.; Růžička K.; Shaw J. M. Heat capacities of tetracene and pentacene. J. Chem. Eng. Data 2008, 53, 2175–2181. 10.1021/je800382b.

[ref42] TP has 198 degrees of freedom. The maximum value of *C*_*v*_ (= 198*R*) is 1646 J K^–1^ mol^–1^, considering the highest intramolecular vibration energy (Table S3), which corresponds to 4500 K. At room temperature, the value of *C*_*p*_ (∼75*R*) is 624 J K^–1^ mol^–1^, which is not even half of the maximum value. Although large differences between *C*_*p*_ and *C*_*v*_ appear in the temperature region where the anharmonic effects of lattice vibration on *C*_*p*_ becomes significant, such anharmonic effects are expected to be small below 300 K. Furthermore, anharmonic effects are terms related to lattice vibrations, and thus, the contribution of the lattice vibrations to the specific heat of single-crystalline TP (75*R*) is only 6*R*. Judging from the above, the difference between *C*_*p*_ and *C*_*v*_ is assumed to be small.

[ref43] In principle, Einstein temperatures originating from intramolecular vibrations can be obtained from theoretical calculations. Our attempts to fit the experimentally obtained *C*_*p*_ curve using the equation of *C*_*v*_ with Einstein temperatures (θ_*Ei*_, *i* = 1–192) obtained by DFT calculations, together with two Debye temperatures (θ_D1_ and θ_D2_) as parameters, resulted in a slight deviation between the experimental and theoretical curves in the low temperature range below 100 K (Figure 2g). This is most likely due to the difference between the molecular structure in the crystal and the computationally optimized one. We set three Einstein temperatures (θ_*E*1_, θ_*E*2_, and θ_*E*3_) and the number of degrees of freedom to be assigned to each, so that the theoretical *C*_*p*_ curve agrees well with the experimental curve over the entire temperature range.

[ref44] Einstein A. Elementary observations on thermal molecular motion in solids. Ann. Phys. 1911, 340, 679–694. 10.1002/andp.19113400903.

[ref45] Iwasaki Y.; Yoshino H.; Kuroda N.; Kikuchi K.; Murata K. Thermal conductivity of molecular crystal with various types of chemical bonding: Quasi-one-dimensional organic superconductor (DMET)_2_AuI_2_. J. Phys. Soc. Jpn. 2015, 84, 054601 10.7566/JPSJ.84.054601.

[ref46] Ashcroft N.; Mermin N. Solid State Physics; Saunders College Publishing: Fort Worth, TX, 1976.

[ref47] Mukhopadhyay S.; Parker D. S.; Sales B. C.; Puretzky A. A.; McGuire M. A.; Lindsay L. Two-channel model for ultralow thermal conductivity of crystalline Tl_3_VSe_4_. Science 2018, 360, 1455–1458. 10.1126/science.aar8072. 29954978

[ref48] Gueye M. N.; Vercouter A.; Jouclas R.; Guérin D.; Lemaur V.; Schweicher G.; Lenfant S.; Antidormi A.; Geerts Y.; Melis C.; Cornil J.; Vuillaume D. Thermal conductivity of benzothieno-benzothiophene derivatives at the nanoscale. Nanoscale, Nanoscale 2021, 13, 3800 10.1039/D0NR08619C. 33565562

[ref49] Shao C.; Shiomi J. Negligible contribution of inter-dot coherent modes to heat conduction in quantum-dot superlattice. Materials Today Physics 2022, 22, 100601 10.1016/j.mtphys.2021.100601.

[ref50] Taylor R. The thermal conductivity of pyrolytic graphite. Philos. Mag. 1966, 13, 157–166. 10.1080/14786436608211993.

[ref51] Chiritescu C.; Cahill D. G.; Nguyen N.; Johnson D.; Bodapati A.; Keblinski P.; Zschack P. Ultralow thermal conductivity in disordered, layered WSe_2_ crystals. Science 2007, 315, 351–353. 10.1126/science.1136494. 17170252

