# Supplementary material for: Insights into Thermal Transport through Molecular π-Stacking
Source: J Am Chem Soc. 2023 Sep 27;145(40):22115–21. doi: 10.1021/jacs.3c07921 (PMC10571076; doi:10.1021/jacs.3c07921)
Supplement: Supplementary file 1 — ja3c07921_si_001.pdf [file ja3c07921_si_001.pdf]

Supporting Information

Insights into Thermal Transport through Molecular π-Stacking

Ryosuke Takehara,* Natsuki Kubo, Meguya Ryu, Suguru Kitani, Shusaku Imajo, Yoshiaki Shoji, Hitoshi Kawaji, Junko Morikawa,* and Takanori Fukushima*

*To whom correspondence should be addressed.
E-mail: takehara.r.ab@m.titech.ac.jp (R.T.), morikawa.j.aa@m.titech.ac.jp (J.M.), fukusihma@res.titech.ac.jp (T.F.)

Table of Contents

1. Materials ········· **S2**
2. Methods ········· **S2**
3. Supporting Figures (Figures S1–S6) ········· **S5**
4. Supporting Tables (Tables S1–S4) ········· **S11**
5. Supporting References ········· **S14**

1. Materials

Unless otherwise stated, all commercial reagents were used as received. Triphenylene-2,3,6,7,10,11-hexacarboxylic acid methyl ester (TP) was prepared according to previously reported procedures.[S1]

2. Methods

Single-crystal X-ray analysis. Single crystals of TP, obtained by recrystallization from toluene, were coated with immersion oil (type B: Code 1248, Cargille Laboratories, Inc.) and mounted on a MicroMount (MiTeGen, LLC.). Diffraction data were collected at given temperatures under a cold nitrogen gas stream on a RIGAKU model XtaLAB Synergy-DW diffractometer system equipped with a HyPix-6000 detector, using Cu-$K\alpha$ radiation (λ = 1.54184 Å). Based on the X-ray crystallographic data (see Table S2), the temperature-dependent volume change was evaluated from 93 to 293 K at intervals of 40 K. The density at each temperature was calculated from the molecular weight of TP (MW = 576.51) and the number of molecules in the unit cell (Z = 4). The temperature dependence of the density below 93 K was extrapolated with the quadratic function.[S2]

Crystal data for TP at 93 K: colorless needle, 0.42 × 0.06 × 0.06 mm^3, orthorhombic, $Pna2_1$, a = 6.80100(10) Å, b = 15.4370(2) Å, c = 26.2402(4) Å, V = 2754.88(7) Å^3, Z = 4, density$_{calcd}$ = 1.390 g cm^{-3}, T = 93 K, Cu$K\alpha$ radiation, λ = 1.54184 Å, μ = 0.924 mm^{-1}, R_{int} = 0.0853, GOF = 1.105, R_1 = 0.0454 ($I > 2\sigma(I)$), wR_2 = 0.1171 (all data), $\Delta\rho_{max,min}$ = 0.329, –0.232 eÅ^3.

Crystal data for TP at 133 K: colorless needle, 0.45 × 0.06 × 0.04 mm^3, orthorhombic, $Pna2_1$, a = 6.81920(10) Å, b = 15.44350(10) Å, c = 26.2260(3) Å, V = 2761.92(5) Å^3, Z = 4, density$_{calcd}$ = 1.386 g cm^{-3}, T = 133 K, Cu$K\alpha$ radiation, λ = 1.54184 Å, μ = 0.921 mm^{-1}, R_{int} = 0.0335, GOF = 1.049, R_1 = 0.0322 ($I > 2\sigma(I)$), wR_2 = 0.0862 (all data), $\Delta\rho_{max,min}$ = 0.193, –0.181 eÅ^3.

Crystal data for TP at 173 K: colorless needle, 0.45 × 0.06 × 0.04 mm^3, orthorhombic, $Pna2_1$, a = 6.84080(10) Å, b = 15.4542(2) Å, c = 26.2352(3) Å, V = 2773.56(6) Å^3, Z = 4, density$_{calcd}$ = 1.381 g cm^{-3}, T = 173 K, Cu$K\alpha$ radiation, λ = 1.54184 Å, μ = 0.917 mm^{-1}, R_{int} = 0.0502, GOF = 1.098, R_1 = 0.0328 ($I > 2\sigma(I)$), wR_2 = 0.0921 (all data), $\Delta\rho_{max,min}$ = 0.178, –0.174 eÅ^3.

Crystal data for TP at 213 K: colorless needle, 0.45 × 0.06 × 0.04 mm^3, orthorhombic, $Pna2_1$, a = 6.86680(10) Å, b = 15.4549(2) Å, c = 26.2350(4) Å, V = 2784.21(7) Å^3, Z = 4, density$_{calcd}$ = 1.375 g cm^{-3}, T = 213 K, Cu$K\alpha$ radiation, λ = 1.54184 Å, μ = 0.914 mm^{-1}, R_{int} = 0.0456, GOF = 1.090, R_1 = 0.0350 ($I > 2\sigma(I)$), wR_2 = 0.0977 (all data), $\Delta\rho_{max,min}$ = 0.150, –0.197 eÅ^3.

Crystal data for TP at 253 K: colorless needle, 0.45 × 0.06 × 0.04 mm^3, orthorhombic, $Pna2_1$, a = 6.89050(10) Å, b = 15.4680(2) Å, c = 26.2688(3) Å, V = 2799.79(6) Å^3, Z = 4, density$_{calcd}$ = 1.368 g cm^{-3}, T = 253 K, Cu$K\alpha$ radiation, λ = 1.54184 Å, μ = 0.909 mm^{-1}, R_{int} = 0.0329, GOF = 1.053, R_1 = 0.0331 ($I > 2\sigma(I)$), wR_2 = 0.0952 (all data), $\Delta\rho_{max,min}$ = 0.138, –0.185 eÅ^3.

Crystal data for TP at 293 K: colorless needle, 0.45 × 0.06 × 0.04 mm^3, orthorhombic, $Pna2_1$, a

= 6.91370(10) Å, b = 15.4718(2) Å, c = 26.2858(3) Å, V = 2811.72(6) Å^3, Z = 4, density$_{calcd}$ = 1.362 g cm^{-3}, T = 293 K, Cu$K\alpha$ radiation, λ = 1.54184 Å, μ = 0.90 5 mm^{-1}, R_{int} = 0.0255, GOF = 1.050, R_1 = 0.0335 ($I > 2\sigma(I)$), wR_2 = 0.0935 (all data), $\Delta\rho_{max,min}$ = 0.127, –0.178 eÅ^3.

Specific heat measurements. Thin cylindrical pellets of single-crystalline TP were prepared by compression molding. Using the pellet samples, the temperature dependence of specific heat was measured on a Quantum Design Physical Properties Measurement System (PPMS) from 2 to 300 K based on a standard relaxation method. The specific heat of TP was also evaluated for its powder samples based on an enthalpy method using a PerkinElmer model 8500 compensated differential scanning calorimeter at a temperature range from 200 to 300 K.

Thermal diffusivity measurements using a micro-temperature wave analysis (μTWA). Using thermal deposition, a sensor (serving as a thermocouple) of Au-Ni and a heater circuit of Au were prepared on SiO_2 and indium-tin-oxide (ITO) substrates, respectively. A single crystal of TP, the crystallographic face of which was determined in advance by single-crystal X-ray analysis, was cut to fit the size of the measuring device (~30×30×50 μm^3) and then sandwiched between the sensor and heater arranged to measure thermal diffusivity (α) in the direction of $\pi_{//}$ or $\pi_{\perp}$, *i.e.*, $\alpha_{//}$ and $\alpha_{\perp}$, respectively. Alternating current (AC) voltage was applied to the heater circuit by a function generator, causing periodic Joule heating at one side of the sample. The temperature change at the other side of the sample was detected as a voltage change of the thermocouple. For low-temperature thermal diffusivity measurements under vacuum, the sample was fixed with an epoxy resin (Stycast 2870FT) to ensure the contact to the circuit. The value of α at 296 K obtained for a sample without fixing by the resin under ambient conditions was confirmed to be comparable to that for a sample fixed with the resin under vacuum (Figure S1b, green filled circle). A negligible effect of the resin was also confirmed by the fact that the value and behavior of thermal diffusivity for the resin alone (Figure S1b, black crosses) is very different from those for TP measured under identical conditions (Figure S1b, red filled circles).

At a given sample temperature, the phase delay ($\Delta\theta$) of the output signal from the sensor against the input voltage to the heater circuit is represented by the following equation:

$$\Delta\theta = -\sqrt{\frac{\pi f}{\alpha}}d - \frac{\pi}{4}$$

where f is a lock-in frequency, and d is the sample length in the measurement direction. The value of α at the sample temperature can be obtained from the slope of a $\Delta\theta$–$f^{1/2}$ plot. The values of $\alpha_{//}$ and $\alpha_{\perp}$ at 296 K were measured for eight independent samples (see Table S1), and the average value with confidence intervals of 95% was obtained (Figures 2b,e,f, and 3). The temperature dependence of α was measured between 9 and 300 K. The validation of the μTWA technique to

measure thermal diffusivity has already been shown through measurements of crystalline sapphire and borosilicate glass (BK7)[S3].

Ultrasonic measurements. $LiNbO_3$ piezoelectric transduces were bonded to both ends of a single-crystal sample of TP. A vibration of 38 MHz was applied to one side of the sample to generate longitudinal sound waves in the crystal, and an echo signal was detected at the other side (Figure S5). The sound velocity was determined using the length of the sample and the time of arrival of the echo signal.

Density functional theory (DFT) calculations. DFT calculations were performed using the Gaussian 16 program package.[S4] The geometry optimization of TP in vacuum was carried out using the ωB97XD hybrid functional with a basis set of 6-31G(d). Cartesian coordinates and energy of the computed structure are listed in Table S4.

Derivation of the anisotropic Einstein thermal conduction model.

Since the Einstein thermal conduction model[S5, S6] holds true for structurally isotropic materials, we modified it as follows, so that the model can be applied to anisotropic materials such as single-crystalline TP.

First, the equation for $C_{v\mathrm{E}}$ (page 4, left column, line 1) in the main text is modified to give

$$C_{v\mathrm{E}} = 3nk_{\mathrm{B}}\left(\frac{\theta_{\mathrm{E}}}{T}\right)^2 \frac{e^{\theta_{\mathrm{E}}/T}}{(e^{\theta_{\mathrm{E}}/T}-1)^2},$$

where n is the number density of molecules. This equation expressing the specific heat per volume is substituted into the equation for thermal conductivity, $\kappa = C_{v\mathrm{E}} v_{\mathrm{th}} l/3$, to obtain

$$\kappa = nk_{\mathrm{B}}\left(\frac{\theta_{\mathrm{E}}}{T}\right)^2 \frac{e^{\theta_{\mathrm{E}}/T}}{(e^{\theta_{\mathrm{E}}/T}-1)^2} v_{th} l,$$

where l and v_{th} are the mean free path and velocity of thermal carriers, respectively. In the Einstein thermal conduction model, l and v_{th} are given as $l = u$ and $v_{\mathrm{th}} = u/\tau$, where u and τ are the center-to-center intermolecular distance and the scattering time, respectively, and τ corresponds to one-half period of molecular vibration, leading to the relationship, $\tau = \hbar\omega/k_{\mathrm{B}}\theta_{\mathrm{E}}$. As a result, the Einstein thermal conduction model describing anisotropic materials can be expressed as

$$\kappa = \frac{k_{\mathrm{B}}^2}{\hbar}\frac{nu^2}{\pi}\theta_E \frac{\left(\frac{\theta_{\mathrm{E}}}{T}\right)^2 e^{\theta_{\mathrm{E}}/T}}{(e^{\theta_{\mathrm{E}}/T}-1)^2}.$$

3. Supporting Figures

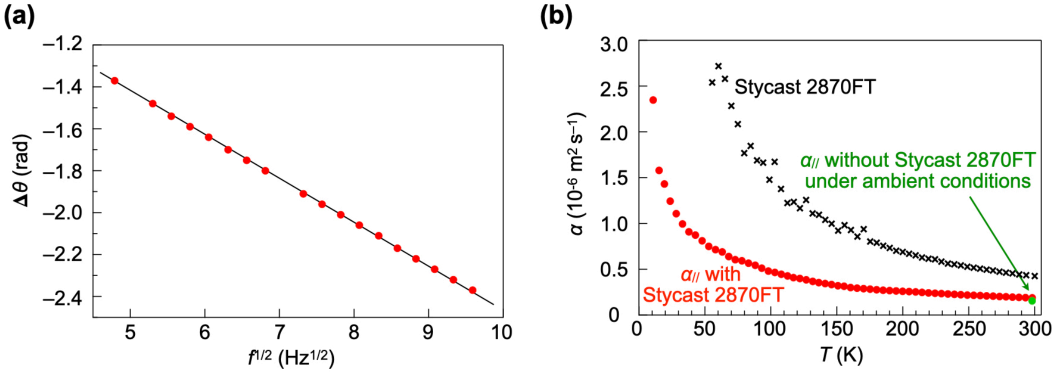

Figure S1. (a) Plots of $\Delta\theta$ against $f^{1/2}$ in the $\pi_{//}$ direction at 296 K. (b) Temperature dependence of the thermal diffusivity of a single crystal of TP ($\alpha_{//}$) with Stycast 2870FT (red filled circles) and Stycast 2870FT alone (black crosses). The value of $\alpha_{//}$ at 298 K (green filled circle) of a single crystal of TP ($\alpha_{//}$) in the absence of Stycast 2870FT is shown for reference, and is almost identical to that obtained in the presence of Stycast 2870FT. Furthermore, given the fact that Stycast 2870FT exhibits much higher thermal diffusivity than single-crystalline TP over the whole temperature range measured, the influence of Stycast 2870FT on the measurements, if any, is considered to be negligible.

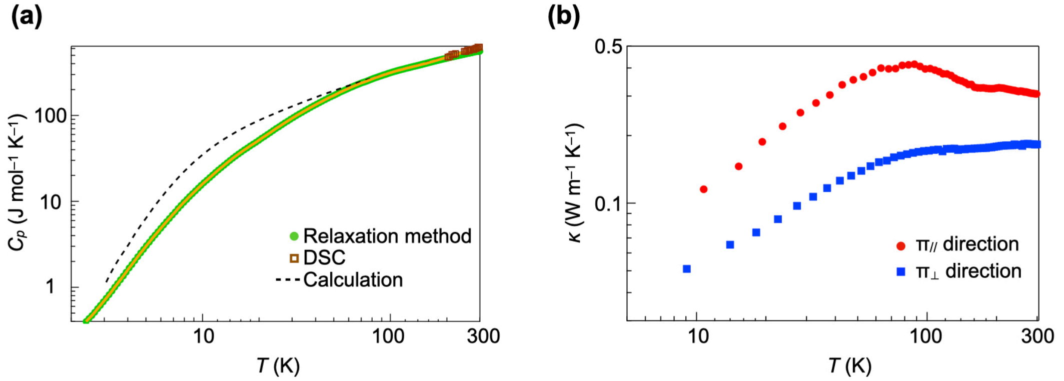

Figure S2. Log-log plots of the data presented in (a) Figure 2c and (b) Figure 2e.

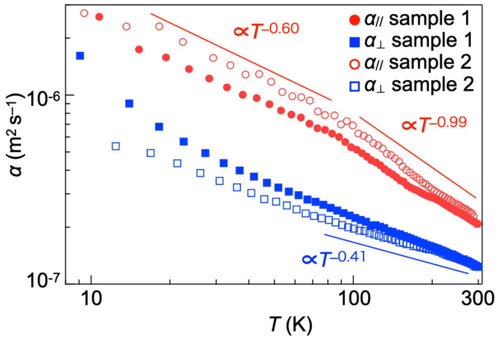

Figure S3. Temperature dependence of $\alpha_{//}$ and $\alpha_{\perp}$ of single-crystal samples of TP. The data for sample 1 (red filled circles) correspond to those provided in Figure 2f. The slopes for sample 2 are given here, while those for sample 1 are presented in Figure 2f.

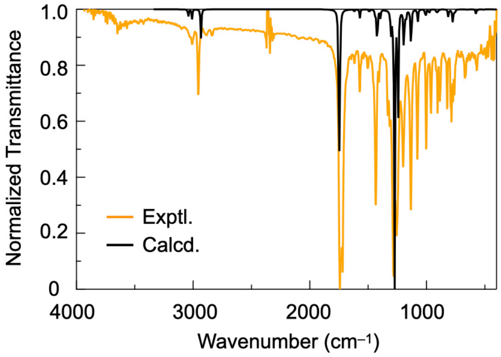

Figure S4. IR spectrum (KBr) of TP at 298 K and the vibrational energy profile of TP calculated at the *ω*B97XD/6-31G(d) level. The computationally simulated profile is in good agreement with the experimental spectrum with respect to the peak positions. Multiplying the calculated values by a factor of 0.96[S7] and then converting them to units of temperature gives the Einstein temperatures (θ_E) that correspond to the intramolecular vibrations (see Table S3).

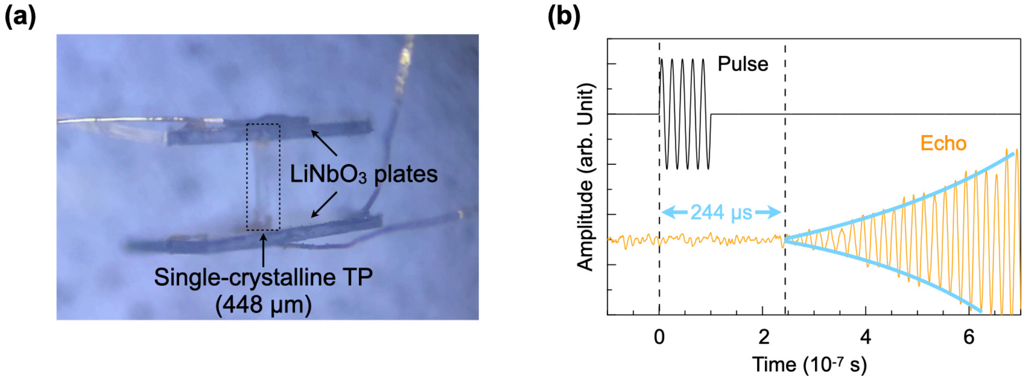

Figure S5. (a) Photograph of a 448 μm-long single-crystal sample of TP sandwiched by two $LiNbO_3$ plates. (b) Applied input pulse and observed echo signal in the ultrasonic measurement. When longitudinal mechanical waves with a frequency of 38 MHz were applied to the sample from one side, the echo signal was detected after 244 μs, which corresponds to the time that it takes for the wave to transfer edge to edge (448 μm) within the sample. Thus, the sound velocity was calculated to be 1840 m^{-1} s. An independent experiment using a different single-crystal sample with a length of 646 μm gave a sound velocity of 1960 m^{-1} s, confirming the reproducibility of the value within an empirical error (±10%) for ultrasonic measurements. We used the average value of these (1900 m^{-1} s) for evaluating MFPs.

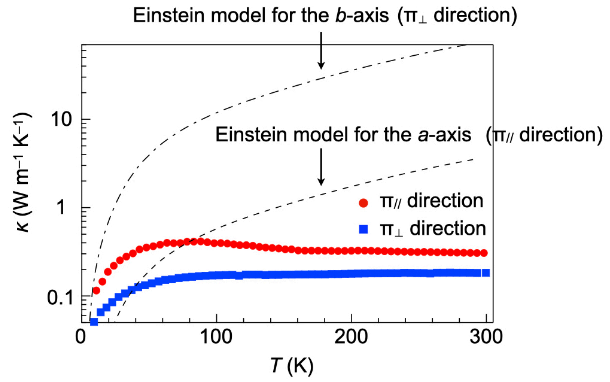

Figure S6. Calculated temperature dependence of the thermal conductivities (black broken curves) for the *a*-axis ($\pi_{//}$ direction) and *b*-axis ($\pi_{\perp}$ direction) using the Einstein thermal conductivity model, from which the data below 0.5 W m^{-1} K^{-1} are extracted and shown in Figure 2e. The calculated thermal conductivities deviate largely from the experimental data (red filled circles and blue filled squares).

4. Supporting Tables

Table S1. $\alpha_{//}$ and $\alpha_{\perp}$ of eight single-crystal samples of TP

Sample No.	$\alpha_{//}$ (10^{-7} m^2 s^{-1})	$\alpha_{\perp}$ (10^{-7} m^2 s^{-1})
#1	2.384	1.158
#2	1.676	0.881
#3	2.406	1.119
#4	1.775	1.252
#5	2.504	1.749
#6	1.885	1.041
#7	2.322	1.300
#8	1.700	1.388

Table S2. Temperature dependence of lattice constants and unit-cell volumes.

	93 K	133 K	173 K	213 K	253 K	293 K
a (Å)	6.8010(1)	6.8192(1)	6.8408(1)	6.8668(1)	6.8907(1)	6.9121(1)
b (Å)	15.4370(2)	15.4435(1)	15.4542(2)	15.4549(2)	15.4531(2)	15.4692(3)
c (Å)	26.2402(4)	26.2260(3)	26.2352(3)	26.2350(4)	26.2431(4)	26.2820(4)
V (Å^3)	2754.88(7)	2761.92(5)	2773.56(6)	2784.21(7)	2794.44(7)	2810.19(8)

Table S3. Einstein temperatures of 192 intramolecular vibrational modes in single-crystalline TP estimated by DFT calculations.

No.	*E* (K)	No.	*E* (K)	No.	*E* (K)	No.	*E* (K)
1	21.0104591	49	522.438091	97	1432.96274	145	2064.12706
2	21.3153429	50	534.897022	98	1457.55209	146	2064.42255
3	30.9982703	51	543.156928	99	1457.65791	147	2064.94584
4	38.1912902	52	561.178641	100	1554.11171	148	2078.06068
5	39.1933868	53	566.014786	101	1554.32956	149	2078.3541
6	44.0962555	54	616.891702	102	1569.64324	150	2078.88872
7	49.2426721	55	666.901073	103	1635.28015	151	2081.06711
8	75.9497753	56	676.796604	104	1636.61255	152	2081.2956
9	76.4860503	57	698.908624	105	1640.18302	153	2081.30265
10	91.9335887	58	739.166686	106	1641.84821	154	2105.79157
11	93.5990528	59	741.328916	107	1642.23653	155	2153.79758
12	100.797598	60	814.497438	108	1642.34912	156	2155.75646
13	104.103661	61	833.237086	109	1642.62292	157	2206.18068
14	108.708967	62	833.699454	110	1643.01124	158	2271.94592
15	126.320946	63	902.667156	111	1660.59463	159	2272.01071
16	135.759911	64	921.288691	112	1694.15201	160	2340.56937
17	138.24374	65	926.278314	113	1694.74286	161	2341.45487
18	156.880609	66	936.466986	114	1696.89887	162	2343.85057
19	174.346431	67	951.833296	115	1700.09911	163	2521.75545
20	180.03824	68	957.150044	116	1700.7256	164	2522.42559
21	180.911725	69	1008.50549	117	1717.18297	165	2523.99463
22	190.741085	70	1029.85413	118	1719.5969	166	2532.14319
23	194.081959	71	1039.60861	119	1725.76212	167	2532.40621
24	198.089931	72	1041.43819	120	1725.87499	168	2532.97233
25	201.072736	73	1093.77474	121	1798.48085	169	4237.63337
26	208.442304	74	1094.72282	122	1798.79029	170	4238.97447
27	214.178043	75	1117.25051	123	1801.84065	171	4239.47897
28	231.711142	76	1119.7733	124	1823.35105	172	4239.70553
29	239.418057	77	1121.1057	125	1840.73219	173	4239.82655
30	241.277199	78	1127.88401	126	1841.75584	174	4240.0578
31	269.214751	79	1135.96447	127	1862.79185	175	4349.23549
32	273.245931	80	1137.33858	128	1864.67531	176	4349.32031
33	277.850547	81	1159.5444	129	1866.9609	177	4349.44008
34	307.561868	82	1175.04872	130	1884.9554	178	4349.48732
35	313.721709	83	1177.08358	131	1889.63489	179	4350.84984
36	323.580217	84	1191.81747	132	1947.75815	180	4351.6403
37	330.582046	85	1202.75143	133	2012.75656	181	4393.57474
38	353.040806	86	1284.55782	134	2016.42898	182	4394.37142
39	360.04595	87	1295.02886	135	2016.60429	183	4394.57463
40	400.306085	88	1297.0173	136	2051.96901	184	4396.7975
41	407.644709	89	1313.23057	137	2052.7353	185	4397.49775
42	414.85776	90	1316.0943	138	2054.50906	186	4398.14316
43	438.926451	91	1316.52296	139	2055.10143	187	4466.55912
44	462.180507	92	1327.23975	140	2056.59739	188	4470.12461
45	467.211987	93	1327.65515	141	2056.90573	189	4472.64063
46	492.661428	94	1408.29381	142	2061.75016	190	4501.93835
47	500.349003	95	1413.317	143	2062.9288	191	4505.71645
48	501.681126	96	1413.84927	144	2063.77949	192	4509.33554

Table S4. Optimized Cartesian coordinates of TP (in Å) at the *ω*B97XD/6-31G(d) level (–2059.969422 hartree).

	Atom	X	Y	Z	No.	Atom	X	Y	Z
1	O	-1.363789	5.866807	-0.781928	34	H	2.805082	2.12278	0.01012
2	O	0.583899	5.534021	0.270251	35	C	1.327139	0.545356	0.004163
3	O	-4.257057	3.785975	-0.766447	36	C	0.161197	1.424937	-0.042572
4	O	-3.300077	4.796476	0.993229	37	C	-0.585693	5.132694	-0.233521
5	O	-4.324625	-4.114345	-1.03461	38	C	0.88359	6.918695	0.104938
6	O	-5.095829	-2.264791	-0.036521	39	H	0.908289	7.180353	-0.951513
7	O	-1.07179	-5.599164	-0.639498	40	H	1.858838	7.06492	0.557874
8	O	-2.619514	-5.222399	0.940247	41	H	0.134903	7.531071	0.604324
9	O	5.721051	-1.727843	0.976139	42	C	-3.32535	3.937692	-0.024758
10	O	4.519062	-3.272255	-0.111034	43	C	-4.396836	5.705414	1.057947
11	O	5.381966	1.849818	0.7131	44	H	-4.406788	6.336	0.170402
12	O	5.846708	0.381845	-0.918443	45	H	-4.231519	6.304497	1.94771
13	C	0.29874	2.81871	-0.052477	46	H	-5.339888	5.166552	1.128869
14	H	1.277098	3.269799	-0.051265	47	C	-4.124511	-3.075337	-0.464363
15	C	-0.787577	3.662083	-0.078818	48	C	-6.425722	-2.693932	-0.32295
16	C	-2.077001	3.11268	-0.076477	49	H	-6.571407	-2.793301	-1.397182
17	C	-2.226926	1.748436	-0.092543	50	H	-7.076759	-1.925303	0.080816
18	H	-3.23482	1.364563	-0.105146	51	H	-6.625723	-3.65265	0.152259
19	C	-1.129662	0.876977	-0.081285	52	C	-1.749764	-4.845667	0.003837
20	C	-1.307266	-0.573192	-0.094937	53	C	-2.866858	-6.62429	1.021116
21	C	-2.579649	-1.15053	-0.194694	54	H	-3.303393	-6.976852	0.088033
22	H	-3.453557	-0.528332	-0.294841	55	H	-3.566714	-6.753675	1.840496
23	C	-2.767651	-2.51295	-0.198739	56	H	-1.944078	-7.167494	1.216323
24	C	-1.652618	-3.354603	-0.084176	57	C	4.728269	-2.047842	0.378757
25	C	-0.39719	-2.803545	-0.020629	58	C	5.55684	-4.219613	0.133909
26	H	0.435768	-3.486505	0.034462	59	H	6.488007	-3.888464	-0.322435
27	C	-0.1892	-1.417723	-0.027017	60	H	5.220799	-5.147657	-0.317156
28	C	1.154779	-0.846812	0.027499	61	H	5.712361	-4.347376	1.203788
29	C	2.290795	-1.662398	0.106473	62	C	5.075749	0.910839	0.030502
30	H	2.190013	-2.733875	0.156425	63	C	7.184401	0.872873	-0.968836
31	C	3.564237	-1.143847	0.141696	64	H	7.193434	1.949806	-1.126573
32	C	3.735636	0.245844	0.080284	65	H	7.655319	0.36102	-1.801977
33	C	2.630704	1.058631	0.030753	66	H	7.699094	0.639503	-0.038193

5. Supporting References

S1. Osawa, T.; Kajitani, T.; Hashizume, D.; Ohsumi, H.; Sasaki, S.; Takata, M.; Koizumi, Y.; Saeki, A.; Seki, S.; Fukushima, T.; Aida, T. Wide-range 2D lattice correlation unveiled for columnarly assembled triphenylene hexacarboxylic esters, *Angew. Chem. Int. Ed.* **2012**, *51*, 7990–7993.

S2. Endo, S.; Watanabe, Y.; Sasaki, T.; Fukase, T.; Toyota, N. Temperature dependence of lattice parameters of α-(BEDT-TTF)$_2$MHg(XCN)$_4$ (M=K, Rb, NH_4, and X=S, Se), *Synth. Met.* **1997**, *86*, 2013-2014.

S3. Morikawa, J.; Orie, A.; Hashimoto, T.; Juodkazis, S. Thermal and optical properties of the femtosecond-laser-structured and stress-induced birefringent regions in sapphire, *Optics Express*, **2010**, *18*, 8300.

S4. Gaussian 16, Revision C.01, Frisch, M. J.; Trucks, G. W.; Schlegel, H. B.; Scuseria, G. E.; Robb, M. A.; Cheeseman, J. R.; Scalmani, G.; Barone, V.; Petersson, G. A.; Nakatsuji, H.; Li, X.; Caricato, M.; Marenich, A. V.; Bloino, J.; Janesko, B. G.; Gomperts, R.; Mennucci, B.; Hratchian, H. P.; Ortiz, J. V.; Izmaylov, A. F.; Sonnenberg, J. L.; Williams-Young, D.; Ding, F.; Lipparini, F.; Egidi, F.; Goings, J.; Peng, B.; Petrone, A.; Henderson, T.; Ranasinghe, D.; Zakrzewski, V. G.; Gao, J.; Rega, N.; Zheng, G.; Liang, W.; Hada, M.; Ehara, M.; Toyota, K.; Fukuda, R.; Hasegawa, J.; Ishida, M.; Nakajima, T.; Honda, Y.; Kitao, O.; Nakai, H.; Vreven, T.; Throssell, K.; Montgomery, J. A., Jr.; Peralta, J. E.; Ogliaro, F.; Bearpark, M. J.; Heyd, J. J.; Brothers, E. N.; Kudin, K. N.; Staroverov, V. N.; Keith, T. A.; Kobayashi, R.; Normand, J.; Raghavachari, K.; Rendell, A. P.; Burant, J. C.; Iyengar, S. S.; Tomasi, J.; Cossi, M.; Millam, J. M.; Klene, M.; Adamo, C.; Cammi, R.; Ochterski, J. W.; Martin, R. L.; Morokuma, K.; Farkas, O.; Foresman, J. B.; Fox, D. J. Gaussian, Inc., Wallingford CT, 2016.

S5. Einstein, A. Elementary observations on thermal molecular motion in solids, *Ann. Phys.* **1911**, *35*, 679–694.

S6. Iwasaki, Y.; Yoshino, H.; Kuroda, N.; Kikuchi, K.; Murata, K. Thermal conductivity of molecular crystal with various types of chemical bonding: Quasi-one-dimensional organic superconductor (DMET)$_2$AuI$_2$, *J. Phys. Soc. Jpn.* **2015**, *84*, 054601.

S7. Ando, S.; Harada, M.; Okada, T.; Ishige, R. Effective reduction of volumetric thermal expansion of aromatic polyimide films by incorporating interchain crosslinking, *Polymers* **2018**, *10*, 761.
